# Effects of Danggui Buxue decoction on host gut microbiota and metabolism in GK rats with type 2 diabetes

**DOI:** 10.3389/fmicb.2022.1029409

**Published:** 2022-10-24

**Authors:** Wen-kai Wang, Lu Fan, Fan Ge, Zihang Li, Jingtian Zhu, Kai Yin, Jinyan Xia, Mei Xue

**Affiliations:** ^1^College of Traditional Chinese Medicine, College of Integrated Chinese and Western Medicine, Nanjing University of Chinese Medicine, Nanjing, China; ^2^Shuguang Hospital, Shanghai University of Traditional Chinese Medicine, Shanghai, China; ^3^School of Medicine and Holistic Integrative Medicine, Nanjing University of Chinese Medicine, Nanjing, China

**Keywords:** Danggui Buxue decoction, type 2 diabetes mellitus, gut microbiota, metabolism, inflammation, oxidative stress, isoflavone, traditional Chinese medicine

## Abstract

Type 2 diabetes mellitus (T2DM) is a chronic metabolic disorder characterized by persistent abnormally elevated blood sugar levels. T2DM affects millions of people and exerts a significant global public health burden. Danggui Buxue decoction (DBD), a classical Chinese herbal formula composed of *Astragalus membranaceus* (Huangqi) and *Angelica sinensis* (Danggui), has been widely used in the clinical treatment of diabetes and its complications. However, the effect of DBD on the gut microbiota of individuals with diabetes and its metabolism are still poorly understood. In this study, a T2DM model was established in Goto-Kakizaki (GK) rats, which were then treated with a clinical dose of DBD (4 g/kg) through tube feeding for 6 weeks. Next, we used 16S rRNA sequencing and untargeted metabolomics by liquid chromatography with mass spectrometry (LC–MS) to detect changes in the composition of the microbiota and cecal metabolic products. Our data show that DBD mediates the continuous increase in blood glucose in GK rats, improves insulin sensitivity, reduces expression of inflammatory mediators, and improves systemic oxidative stress. Moreover, DBD also improves microbial diversity (e.g., *Romboutsia*, *Firmicutes,* and *Bacilli*) in the intestines of rats with T2DM. Further, DBD intervention also regulates various metabolic pathways in the gut microbiota, including alanine, aspartate, and glutamate metabolism. In addition, arginine biosynthesis and the isoflavone biosynthesis may be a unique mechanism by which DBD exerts its effects. Taken together, we show that DBD is a promising therapeutic agent that can restore the imbalance found in the gut microbiota of T2DM rats. DBD may modify metabolites in the microbiota to realize its antidiabetic and anti-inflammatory effects.

## Introduction

Diabetes mellitus is a complex chronic disease which poses a significant global public health issue. Data from the WHO suggest that 422 million people suffer from this disease, and individuals with type 2 diabetes mellitus (T2DM) account for 90% of that (World Health Organization, 2022). The majority of T2DM patients often suffer from various complications as the disease progresses. These include diabetic cerebral microvascular complications, diabetic nephropathy, and cognitive dysfunction (Chatterjee et al., 2017; Van Sloten et al., 2020). These complications negatively affect the quality of life of people with T2DM and increase the burden on public health systems. Recent studies have shown that in the early stages of T2DM, chronic systemic inflammatory responses and oxidative stress, which are responsible for metabolic abnormalities, can harm the retinal vasculature, endothelial cells, and autonomic nerves (Singh et al., 2011; Verrotti et al., 2014; Hammes, 2018). Therefore, there is an urgent need to develop novel strategies to prevent and treat T2DM. Moreover, the development of antidiabetic drugs would also be highly beneficial.

The gut microbiota is associated with diseases of the digestive system, mental illness, and metabolic disorders among others (Zeng et al., 2020; Hackam and Sodhi, 2022; Metwaly et al., 2022). The gut microbiota is implicated in various pathologies *via* its influence on the digestion and absorption of food, production of endogenous metabolites, and competition for growth between pathogenic bacteria (Adak and Khan, 2019). Clinical studies have shown that the gut microbiota of people with diabetes has an unbalanced *Firmicutes*/*Bacteroidetes* ratio and reduced levels of butyrate-producing bacteria (Tilg and Moschen, 2014). In addition, with the change in bacterial strains, changes in indoxyl sulfate, p-cresol sulfate, butyrate, and some other short-chain fatty acids (SCFAs) can also dysregulate immune responses, causing inflammation, oxidative stress, and insulin resistance, which are all characteristics of T2DM, cardiovascular disease, inflammatory bowel disease, autoimmune disease, and a variety of cancers (Yoo et al., 2020). As a key factor in the development of these diseases, there is evidence to show that the intestinal flora can be affected by Chinese traditional herb formulae and pharmacodynamic monomers, which can also induce a therapeutic effect on disease (Feng et al., 2019; Lin et al., 2022). By determining the similarity of 16S rRNA gene sequences, we can divide bacteria into different species to judge gut microbiota diversity. In addition, the impact of bacteria on the body will also be directly reflected at the level of intestinal metabolites. Untargeted metabolomics by liquid chromatography with mass spectrometry (LC–MS), a sensitive, precise, and efficient testing method, is often used to identify metabolites and low-molecular-weight compounds in samples.

Danggui Buxue decoction (DBD), composed of *Astragalus membranaceus* (Huangqi) and *Angelica sinensis* (Danggui) in 5:1 ratio mix, is a well-known traditional Chinese medicine (TCM) formula from the Jin dynasty. DBD has been proven to have significant effects on promoting hematopoiesis, regulating immunity, and protecting cardiac and cerebral vessels (Yang et al., 2021). As a result, DBD has been widely used in clinical practice to treat diabetic nephropathy, female menopausal symptoms, and anemia (Hong et al., 2017; Lin et al., 2017). Preliminary experimental data obtained in our laboratory demonstrated that DBD also reduces blood sugar levels in diabetic rats, improves the symptoms of diabetic nephropathy, and protects and remodels hippocampal neurons in rats (Xue et al., 2020; Wang et al., 2021).

Herein, we used 16S rRNA gene sequence analysis and LC–MS untargeted metabolomics detection to study changes in the cecum microbiota and metabolomics of T2DM GK rats. Concurrently, we also tested inflammatory factors (e.g., indicators of oxidative stress). Subsequently, correlation analysis was used to forecast and investigate the relationship between the gut microbiota, metabolism of intestinal contents, chronic systemic inflammation, and oxidative stress. This study will aid our understanding of the mechanisms by which DBD effectively exerts its anti-inflammatory and antioxidant effects. Moreover, this work will help develop therapeutic strategies that use DBD for early prevention and treatment of diabetes.

## Materials and methods

Methanol, acetonitrile, and formic acid were of LC–MS grade, and 2-propanol was high-performance liquid chromatography (HPLC) grade. These chemicals were all purchased from Thermo Fisher Scientific Inc (Waltham, Massachusetts, United States). The ferulic acid (MW: 194.19, LOT: MUST-17010908, purity ≥98% HPLC)，caffeic acid (MW: 180.15, LOT: MUST-17010908, purity ≥98% HPLC), calycosin (MW: 284.26, LOT: MUST-14070923, purity ≥98% HPLC), and formononetin (MW: 268.26, LOT: MUST-14091205, purity ≥98% HPLC) were provided by Chengdu Must Bio-Technology Co. Ltd., Ligustilide (MW: 284.26, LOT: wkq-00246, purity ≥98% HPLC LOT: wkq-00246) was obtained from Sichuan Weikeqi Biological Technology Co., Ltd. Rat SOD, MDA, TNF-α, IL-1β, IL-18, IL-6 and insulin ELISA Kits and ROS kit were all purchased from Shanghai Enzyme-linked Biotechnology Co., Ltd.

Danggui (the root of *A. sinensis (Oliv.) Diels*, Place of Origin: Gansu Province, Batch number: 259200103) and Huangqi (the root of *Astragalus mongholicus Bge,* Place of Origin: Inner Mongolia, Batch number: 190919001) were purchased from Beijing Tong Ren Tang Nanjing Pharmacy (Han Zhong Road, Nanjing, China). After purchase, the herbs were maintained in closed, lucifugal, and dry conditions at 23 ± 2°C.

### Preparation and composition determination of DBD

The extraction and phytochemical analysis method was in accordance with that reported previously (Wang et al., 2021). Briefly, Huangqi and Danggui (5:1) were soaked for 1 h, then heated to boil and held for 30 min. Following filtration with absorbent gauze, the herbal mixture was again soaked in boiling for 30 min. The extracts were combined, freeze-dried (raw herb: lyophilized powder ≈40.55%), and stored at −80°C until further use. Phytochemical analyses of the composition of DBD extracts were performed using ultra-performance liquid chromatography with photodiode array (UPLC-PDA) (Waters, Milford, United States).

### Animal and experimental design

Healthy specific pathogen-free (SPF) -grade Wistar and Goto-Kakizaki (GK) rats (male, 250–300 g) were obtained from the Shanghai SLAC Laboratory Animal Co., Ltd., Shanghai, China [SCXK (Hu)2017–0005)] and reared in SPF level barrier environmental facilities in the laboratory animal center of Nanjing University of Chinese Medicine. Animals were kept with a 12 h light/dark cycle and with *ad libitum* access to food (sterilization by cobalt radiation) and distilled water. Air conditions was controlled at a temperature of 22–25°C, and 50–60% relative humidity. This study was approved by the Committee on the Ethics of Animal Experiments of Nanjing University of Chinese Medicine and Experimental Animal Welfare of Nanjing University of Chinese Medicine, China (NO. 012071002558). All work detailed here was performed in accordance with Chinese institutional guidelines and ethics.

All animals sustained adaptive feeding for 1 week and then the Wistar rats in the control group, and the GK rats in the model group and DBD group (*n* = 6 for each group) were fed with a high-fat diet for 6 weeks to induce the onset of diabetes. This diet contained 43.5% basic feed, 17.5% lard oil, 12% saccharose, 13% casein, and 10% whole milk powder (Jiangsu Xietong Pharmaceutical Bio-engineering Co., Nanjing, Jiangsu, China). Before administration, DBD lyophilized powder was weighed according to the yield and dissolved in warm water at 30°C to make a solution containing 0.4 g/ml of DBD crude drug. During the 6-week experimental period, control and model group rats were given normal saline *via* intragastric administration, while DBD was administered to rats in DBD group by gavage (10 ml/kg). At the same time, random blood sugar (RBG) of rat tail vein blood was monitored weekly using glucose test papers (Sinocare Inc., Hunan, China).

### Sample collection and determination of biochemical indicators

Following a 24 h fast after the last treatment, all the rats were anesthetized with pentobarbital sodium and euthanized immediately after whole blood collection from the abdominal aorta by vacuum blood collection tube. Next, the whole blood was centrifuged at 4,500 × *g* (centrifuge-SL-16R, Thermo Fisher Scientific Inc., Waltham, Massachusetts, United States) at 4°C for 10 min, and serum biochemical inflammation and oxidative stress indicators, superoxide dismutase (SOD), Malondialdehyde (MDA), tumor necrosis factor-α (TNF-α), interleukin (IL) -1β (IL-1β), IL-18, IL-6, and reactive oxygen species (ROS) were measured according to the manufacturer’s instructions, using commercial kits. The contents of the cecum were also collected and preserved at −80°C for 16S rRNA microbial diversity and LC–MS metabolome analyses.

### Extraction and profiling of intestinal contents metabolites

Next, 50 mg cecal chyme sample was accurately weighed and mixed with 400 μl pre-cooling methanol solution (methanol:water 4:1 ratio). Thus, the mixture was then ground and homogenized by a high-throughput tissue disruptor (Wonbio-96c, Shanghai Wanbai Biotechnology Co., Ltd., Shanghai, China). After vortexing, the sample was sonicated on ice for 10 min (3 times) before incubation at −20°C for 30 min. Finally, after a centrifugation (Centrifuge 5,430 R, Eppendorf AG, Hamburg, Germany) at 13,000 g at 4°C for 15 min, the supernatant was collected for metabolomic analysis. To monitor the stability of the LC–MS during the entire analysis process, a quality control (QC) sample was mixed from all samples in the same volumes. This was used to find variables with large variation in the analysis system and to ensure reliability of the results. The QC sample was injected every eight samples as previously reported (Kirwan et al., 2013; Zhao et al., 2020).

Separation was executed on an ultra HPLC (UHPLC) system (Vanquish Horizon UHPLC System, Thermo Fisher Scientific Inc., Waltham, Massachusetts, United States) equipped with a binary solvent delivery manager and a sample manager, coupled with a Q-Exactive Mass Spectrometer fitted with an electrospray interface (Q-Exactive system, Thermo Fisher Scientific Inc., Waltham, Massachusetts, United States). An ACQUITY BEH C_18_ column (1.7 μm, 2.1 × 100 mm, Waters, Milford, United States) was used at a column temperature of 40°C. The mobile phase consisted of 0.1% formic acid in water (A) and acetonitrile/isopropanol (1:1) (B) under the following gradient: 0–3 min, 5–20% B; 3–9 min, 20–95% B; 9–13 min, 95–95% B; 13–13.1 min, 95–5% B; 13.1–16 min, 5–5% B, and the flow rate was maintained at 0.4 ml/min. The mass spectrometric data were collected using a Thermo UHPLC -Q Exactive Mass Spectrometer equipped with an electrospray ionization (ESI) source operating in either positive or negative ion mode. Specific parameters are as follows: scanning range (*m/z*), 70–1,050 Da; sheath gas flow rate, 40 psi; cone gas flow rate, 10 psi; cone gas heater temp, 400°C; ionspray voltage (ESI+), +3,500 V; ionspray voltage (ESI-), −2,800 V, and the normalized collision energy was 20–40-60 V. Raw data were imported into the Progenesis QI software (Waters Co., Milford, MA, United States) for baseline filtering, peak identification, integration, retention time correction, and peak alignment.

To reduce errors in the experiment and analysis process, the data structure was standardized. The process of data preprocessing was followed as previously reported (Wu et al., 2020): filtering low-quality peaks (>80%), filling in missing values (minimum), normalization, QC sample RSD evaluation (≤30%), and data conversion (Log 10). To reflect the overall difference between samples in each group and the degree of variability between samples within a group, principle component analysis (PCA) was adopted. The orthogonal partial least squares discriminant analysis (OPLS-DA) was used to better distinguish differences between groups, improve the effectiveness and the parsing ability of the model. Moreover, to evaluate the model quality of the OPLS-DA, we performed seven-fold cross validation and response permutation testing. Next, metabolite identification (significant difference between two groups, Student’s *t* test, two-tailed test, *p* < 0.05, variable importance in projection (VIP) > 1) analyses were performed according to mass fragmentation information and matched to some online metabolic databases, including the Human metabolome database (HMDB^1^) and Kyoto Encyclopedia of Genes and Genomes (KEGG^2^). Finally, the classification of differential metabolites in the superclass of the HMDB level, the numbers of differential metabolites in different KEGG pathways, and KEGG topology analysis bubble plot were performed on the Majorbio Cloud Platform.^3^

### Microbial extraction and diversity analysis of cecal contents

Total DNA was extracted from the precise weighing of 100 mg cecal content samples using the E.Z.N.A. soil kit (American BioTek Instruments Co., Ltd., United States) as per manufacturer instructions. The concentration and purity of DNA was determined using a NanoDrop 2000, and DNA quality was assessed using a 1% (w/v) agarose (Yingjie Life Technology Co., Ltd., United States) gel electrophoresis gel. The variable region of V3–V4 was amplified using the 338\u00B0F (5′- ACTCCTACGGGAGGCAGCAG −3′) and 806R (5′- GGACTACHVGGGTWTCTAAT −3′) primers. The amplification procedure was as follows: pre-denaturation at 95°C for 3 min, followed by 27 cycles of denaturation at 95°C for 30 s, annealing for 30 s, and extension at 72°C for 30 s, and the final extension at 72°C for 10 min. PCR products were recovered by agarose gel electrophoresis on a 2% (w/v) gel, which was then purified using an AxyPrep DNA Gel Extraction kit (Axygen, United States), eluted using Tris–HCl, and detected on a 2% (w/v) agarose gel. QuantiFluor™-ST (Promega, United States) was used for quantitative detection. According to the standard operating procedure for the Illumina MiSeq platform (Illumina, United States), a PE 2 × 300 library was constructed. Trimmomatic software was used to control the original sequence, and FLASH software was used to assemble the sequence.

Using UPARSE software (version 7.0^4^) based on 97% similarity, the non-repeated sequences (excluding single sequences) were clustered into operational taxonomic units (OTUs), and the representative sequences of OTUs were obtained. The abundance, evenness, and diversity of sample microbial community were assessed using Alpha diversity analysis. Further, Beta diversity analysis was used to explore similarities or differences in community composition between different grouped samples in hierarchical cluster analysis and principal co-ordinates analysis (PCoA). Subsequently, community composition analysis and linear discriminant analysis (LDA) was performed on samples of different groups to find the species that had significant differences in sample classification using linear discriminate analysis effect size (LEfSe) software. To study the role of the gut microbiome in the intestines, the Phylogenetic Investigation of Communities by Reconstruction of Unobserved States two (PICRUSt2) functional prediction was adopted to study the relative abundance of KEGG function in the bacterial community.

### Statistical analysis

All data are expressed as the mean ± standard deviation (SD). After testing for normal distribution, unpaired Student’s *t*-test between two groups was processed by GraphPad Prism 8.0 (GraphPad Software Inc., San Diego, United States) with two-tailed analysis. Data are considered statistically significant if *p* < 0.05. Significant *p*-values that associated with microbial population and differential metabolite were corrected for multiple hypotheses testing using the Benjamini-Hochberg False Discovery Rate (FDR) method.

## Results

### UPLC analysis of DBD

**Figure 1:**
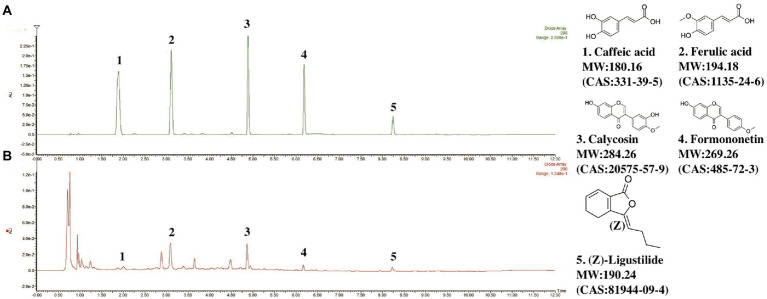
Chromatograms of representative components in DBD. **(A)** Chromatogram of standard mixture of 5 ingredients. **(B)** Chromatogram of DBD. Observation wavelength (λ) = 290 nm.

The UPLC-PDA characteristic chromatogram of DBD is shown in Figure 1. Although DBD consists of only two simple Chinese medicine herbs, it contains a variety of compounds, including organic phenolic acids (caffeic acid, ferulic acid), flavones (formononetin, calycosin), and phthalide (*Z-*Ligustilide). The contents of main representative compounds in DBD have been reported in our previous study (Wang et al., 2021).

### Effect of DBD on insulin resistance and inflammatory biomarkers

**Figure 2:**
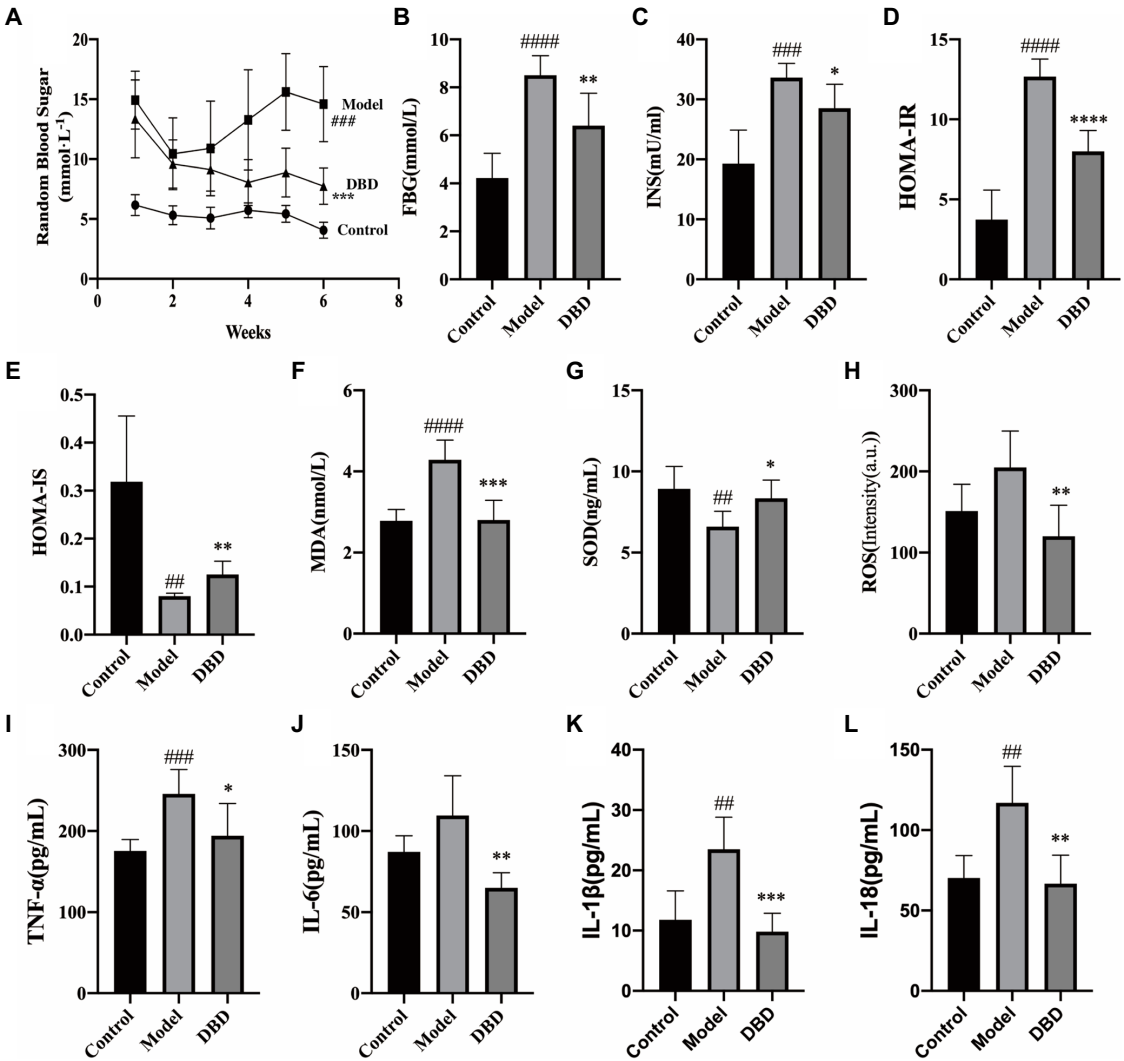
The effects of DBD on the blood sugar, insulin, serum oxidative stress, and inflammation cytokine. **(A)** Random blood sugar of rats at 6 weeks. **(B)** Concentration of the fasting blood-glucose. **(C)** Concentration of the blood serum insulin. **(D)** Levels of HOMA-IR. **(E)** Levels of HOMA-IS. **(F)** Concentration of the blood serum MDA. **(G)** Concentration of the SOD. **(H)** Levels of ROS. **(I)** Concentration of the inflammatory cytokine TNF-α. **(J)** Concentration of IL-6. **(K)** Concentration of the IL-1β. **(L)** Concentration of IL-18. Data are presented as MEAN values ± SD, *n* = 6. Statistical analysis was done by student’s *t* test. Compared to control group ##*p* < 0.01, ###*p* ≤ 0.005, ####*p* ≤ 0.001; compared to model group *p < 0.05, **p ≤ 0.01, ***p ≤ 0.005, ****p ≤ 0.001.

Insulin resistance is one of the significant physiologic changes that occur in early diabetes mellitus and eventually leads to systemic non-infectious chronic inflammation (Ndisang et al., 2017). As shown in Figure 2A, during the 6-week experiment, compared to control group, the RBG of the model and DBD groups was always higher than control group. This may be because GK rats spontaneously developed diabetes. After giving DBD for 3 weeks, the RBG of rats showed a downward trend, and had a significant decrease at the end of the 6-week treatment. Moreover, the high levels of fasting plasma glucose (FBG), insulin (INS), HOMA-IR, and HOMA-IS were reversed by DBD (Figures 2B–E), thereby improving the insulin resistance symptoms in GK rats. To study the systemic oxidative stress situation, we found that the level of antioxidant factor SOD in the serum decreased significantly and the content of oxidation factor MDA and ROS increased, compared to the control group, which were inverted by DBD (Figures 2F–H). The systemic non-infectious chronic inflammation is often accompanied by oxidative stress. Four biochemical parameters in the blood serum indicative of the inflammation were measured in the three groups of rats (Figures 2I–L). The results show that compared to the control group, the serum levels of TNF-α, IL-1β, IL-18, and IL-6 in the model group were upregulated, which indicates a pro-inflammatory state in rats. Interestingly, after 6-weeks of oral DBD, the levels of TNF-α, IL-1β, IL-18, and IL-6 met a remarkable reduced, thereby demonstrating the anti-inflammatory effect of DBD.

### Metabolism analysis

The metabolic makeup of rat cecum contents were analyzed by positive and negative ion modes by LC–MS and the representative total ion chromatograms of the positive and negative different modes of the QC sample are shown in Supplementary Figure 1.

**Figure 3:**
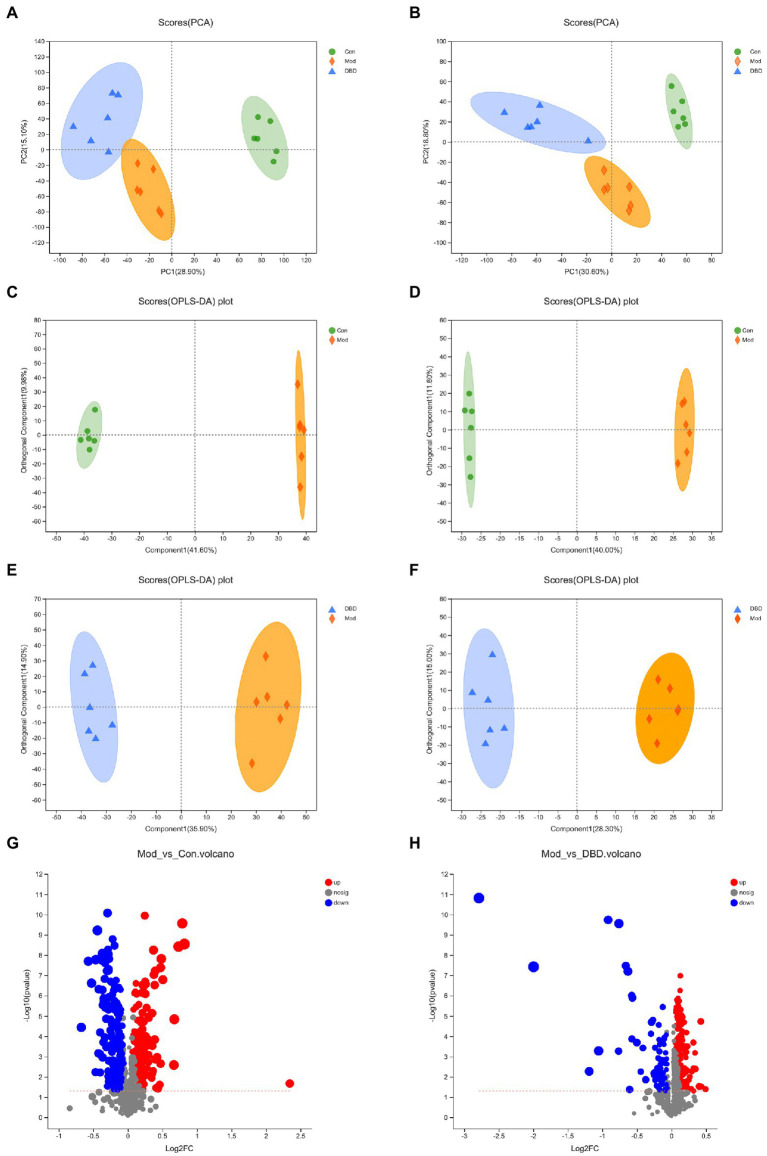
Multivariate statistical analysis of the LC–MS spectra of rat cecal contents taken from the three groups (*n* = 6). **(A)** PCA score map of the three groups in the negative ion model. **(B)** PCA score map of three groups in the positive ion model. **(C)** OPLS-DA score map of control vs. model group in the negative ion model. **(D)** OPLS-DA score map of control vs. model group in the negative ion model. **(E)** OPLS-DA score map of control vs. model group in the negative ion model. **(F)** OPLS-DA score map of control vs. model group in the negative ion model. **(G)** Differential volcanic map of control vs. model group. **(H)** Differential volcanic map of model vs. DBD group.

**Figure 4:**
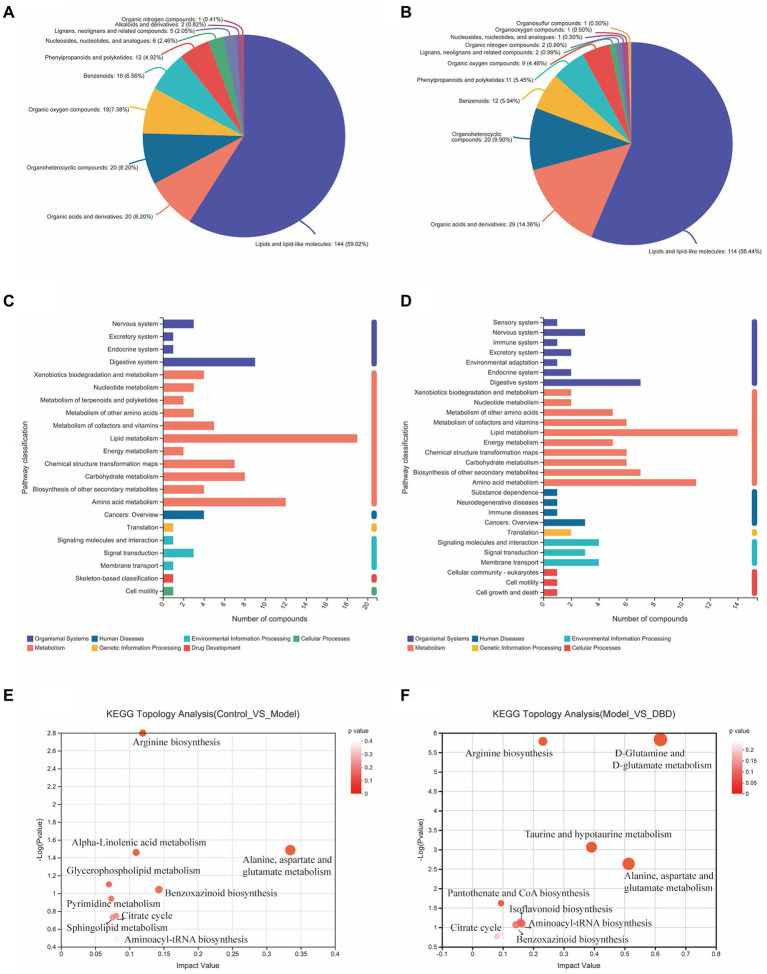
Summary of differential metabolite classification and related pathway analysis. **(A)** Classification of differential metabolite between control and model group in Superclass of HMDB level. **(B)** Classification of differential metabolite between model and DBD group in Superclass of HMDB level. **(C)** Numbers of differential metabolite in different KEGG pathways between control and model group. **(D)** Numbers of differential metabolite in different KEGG pathways between model and DBD group. **(E)** KEGG topology analysis bubble plot between control and model group. **(F)** KEGG topology analysis bubble plot between model and DBD group. Each bubble in the Figure represents a KEGG pathway. The horizontal axis represents the impact value of metabolites in the pathway and the vertical axis represents the enrichment significance of metabolites in the pathway -Log 10 (*p* value). Bubble size represents impact value and the larger the bubble, the more important the pathway is.

PCA is an unsupervised multivariate statistical analysis method that can reflect the overall difference between samples in each group and the degree of variability between samples in the group as a whole. Our data (Figures 3A,B) show that, in both negative and positive ion models, remarkable separation in gastrointestinal contents of the control, model, and DBD groups can be seen, which indicated that the samples in the model group deviated from normal levels and showed metabolic disorders, and in the DBD group, DBD had allowed the abnormal metabolism to gradually recover. Subsequently, in order to discover the similarities and differences between these two groups and to improve the effectiveness and analytical capabilities of the model, Orthogonal Partial Least Squares Discriminant Analyses (OPLS-DA) were performed to identify the potential biomarkers as in Figures 3C–F. Also in order to ensure the reliability of the results, the OPLS-DA model was subjected to 200 permutation test analyses; the intercept of the Q2 regression line was less than 0, and the model was not overfitting (Supplementary Figure 2). Next, volcano plots were adopted to show the upward or downward revision of metabolites between different groups in Figures 3G,H. The metabolites in gastrointestinal contents samples were identified based on MS data, KEGG, and HMDB 4.0. Potential biomarkers were screened according to the VIP value (>1.0) and statistical tests (*p* < 0.05). In total, 479 significantly different metabolites were identified. Compared to the model group, 201 metabolites were significantly downregulated and 70 significantly upregulated in the DBD group. Following intersection of the three groups of data, 62 metabolites were identified as potential biomarkers in both comparisons in the onset and treatment of T2DM. Changes in these potential biomarkers were reversed by DBD (Table 1). To study the potential metabolic pathway related to T2DM and the effect of DBD, HMDB compound classification and KEGG topology analysis based on group-to-group (control vs. model, model vs. DBD) differential metabolites. The HMDB compound classification statistics showed that lipids and lipid-like molecules were the main differential metabolites (Figures 4A,B). Compared to differential metabolites between the control and model groups, the proportion of organic acids and derivatives and organoheterocyclic compounds was higher than that between the control and model group. The KEGG functional pathway statistics (Figures 4C,D) showed that metabolism and organismal systems were the main metabolic pathways of the onset and treatment of T2DM. Finally, based on the identified differential metabolites between the two groups, the KEGG topology analysis was conducted to evaluate the importance of the KEGG pathway for therapy. The results (Figures 4E,F) showed that the occurrence of T2DM and the influence of DBD might be associated with alanine, aspartate, and glutamate metabolism, arginine biosynthesis, benzoxazinoid biosynthesis, Citrate cycle, and aminoacyl-tRNA biosynthesis simultaneously. Further, alpha-Linolenic acid metabolism, glycerophospholipid metabolism, pyrimidine metabolism and sphingolipid metabolism may also took part in the onset of T2DM. The influence of DBD also related to D-Glutamine and D-glutamate metabolism, taurine and hypotaurine metabolism, isoflavonoid biosynthesis, and Pantothenate and CoA biosynthesis.

**Table 1:** Potential biomarkers identified in positive and negative ion mode.

Metabolite	M/Z	Mode	Control vs. Model				Model vs. DBD			
			VIP_pred_OPLS-DA	VIP_PLS-DA	*P* value	Trend	VIP_pred_OPLS-DA	VIP_PLS-DA	*P* value	Trend
(−)-Trans-Carveol glucoside	359.17146	Neg	2.198	2.19	0	↑	1.148	1.113	0.003	↓
(±) 5-iPF2alpha-VI	353.23344	Neg	1.026	1.012	0	↑	1.148	1.105	0	↓
(6E,8R,10Z)-8-hydroxy-3-oxohexadecadienoic acid	281.17574	Neg	1.111	1.088	0.002	↑	1.138	1.117	0.002	↓
(R)-2,3-Dihydro-3,5-dihydroxy-2-oxo-3-indoleacetic acid	222.04079	Neg	1.895	1.912	0	↑	1.057	1.004	0.035	↓
(R)-Shinanolone	234.11241	Pos	1.853	1.846	0	↑	1.183	1.169	0	↓
1,2-Dihydroxyheptadec-16-en-4-yl acetate	311.25736	Pos	1.873	1.873	0	↑	1.227	1.219	0.018	↓
12-OPDA	293.21091	Pos	1.158	1.156	0	↑	1.022	0.984	0.001	↓
19-Oxotestosterone	366.20633	Pos	1.385	1.376	0	↑	1.243	1.193	0.004	↓
2,3-Dinor-6-keto-prostaglandin F1 a	387.203	Neg	2.151	2.146	0	↑	1.165	1.148	0.005	↓
2,5-Dihydroxycinnamic acid methyl ester	433.1147	Neg	1.122	1.128	0.022	↑	1.258	1.15	0.034	↓
25-Hydroxyvitamin D3-26,23-lactol	429.30109	Neg	1.083	1.066	0.003	↑	1.359	1.277	0.006	↓
2-Formaminobenzoylacetate	208.06044	Pos	1.711	1.694	0	↑	1.237	1.185	0	↓
2-Hydroxyquinoline	291.11279	Pos	2.964	2.957	0	↑	1.164	1.086	0.002	↓
3,4-Dimethyl-5-propyl-2-furanheptanoic acid	303.1351	Neg	1.157	1.134	0.004	↑	1.406	1.443	0.001	↓
4-OH-Retinal	345.20964	Neg	1.148	1.137	0	↑	1.385	1.336	0	↓
5-Androstene-3b,16b,17a-triol	351.19107	Pos	1.23	1.223	0.029	↑	1.718	1.742	0.004	↓
5-bromo-3-pheny Salicylic Acid	290.96831	Neg	1.115	1.118	0.01	↑	1.524	1.421	0.01	↓
5-Cis Carbaprostacyclin	349.23862	Neg	1.901	1.882	0	↑	1.61	1.57	0	↓
9,12-Octadecadiynoic Acid	277.21611	Pos	1.125	1.127	0.002	↑	1.201	1.185	0.011	↓
9-OxoODE	295.22665	Pos	1.079	1.083	0.002	↑	1.163	1.15	0.013	↓
Alpha-CEHC	277.14382	Neg	1.578	1.561	0	↑	1.33	1.306	0.001	↓
Auxin b	347.16155	Neg	1.444	1.427	0.002	↑	1.309	1.327	0.004	↓
Blumenol C glucoside	386.22806	Pos	1.361	1.364	0	↑	1.12	1.12	0.005	↓
C75	253.1444	Neg	1.148	1.132	0.001	↑	1.147	1.141	0.002	↓
Carnosic acid	331.19159	Neg	1.518	1.497	0	↑	1.104	1.088	0.005	↓
Cryptomeridiol 11-rhamnoside	385.25995	Neg	1.834	1.822	0	↑	1.535	1.494	0	↓
Cucurbic acid	257.13953	Neg	1.31	1.307	0.003	↑	1.108	1.059	0.033	↓
Cyclo (Leu-Phe)	259.14512	Neg	1.578	1.551	0.002	↑	1.375	1.428	0.004	↓
DG (15:0/18:4(6Z,9Z,12Z,15Z)/0:0)	573.45286	Neg	1.029	1.031	0.008	↑	1.352	1.335	0.006	↓
Falcarinolone	303.16021	Neg	2.352	2.341	0	↑	1.333	1.307	0	↓
Methyl 9,10-epoxy-12,15-octadecadienoate	309.24231	Pos	1.509	1.512	0.001	↑	1.339	1.339	0.021	↓
MG (18:2(9Z,12Z)/0:0/0:0)[rac]	355.28423	Pos	2.051	2.043	0.001	↑	1.536	1.556	0.026	↓
N1,N12-Diacetylspermine	287.24419	Pos	2.137	2.115	0.002	↑	1.767	1.682	0.038	↓
Niazirin	324.10918	Neg	1.014	1.012	0.004	↑	1.204	1.141	0.003	↓
Nomega-Acetylhistamine	154.09744	Pos	1.598	1.613	0.008	↑	2.156	2.146	0.004	↓
O-Demethylfonsecin	297.04044	Neg	2.322	2.318	0	↑	1.4	1.372	0	↓
Physagulin B	583.18265	Neg	1.376	1.377	0.007	↑	1.468	1.465	0.014	↓
Polyethylene, oxidized	243.12366	Neg	1.055	1.049	0	↑	1.186	1.143	0	↓
Pyroglutamylvaline	273.10932	Neg	1.169	1.161	0.008	↑	1.047	1.016	0.033	↓
Quadrone	249.14842	Pos	1.178	1.17	0	↑	1.128	1.115	0	↓
Sterebin E	371.27908	Pos	2.228	2.225	0	↑	1.561	1.57	0.017	↓
(−)-Epicatechin sulfate	184.01223	Neg	1.078	1.087	0.006	↓	2.924	2.969	0	↑
(±)-Enterolactone	297.11309	Neg	1.872	1.875	0	↓	1.382	1.389	0	↑
12 Alpha-hydroxy-3-oxo-5beta-cholan-24-oic Acid	435.27537	Neg	1.082	1.096	0.011	↓	1.187	1.31	0.011	↑
13-HDoHE	337.25237	Pos	1.075	1.089	0.027	↓	1.592	1.672	0.01	↑
17-beta-Estradiol-3-glucuronide	429.19507	Neg	1.783	1.766	0.002	↓	2.032	2.024	0	↑
2’-Hydroxyenterolactone	335.08707	Neg	1.764	1.767	0	↓	1.211	1.23	0	↑
4-(1-hydroxy-3-phenylprop-2-en-1-yl)-5-methoxy-2,6-dimethylbenzene-1,3-diol	299.12885	Neg	1.014	1.011	0.023	↓	1.626	1.631	0	↑
6,7-dihydro-12-epi-LTB4	356.27892	Pos	2.101	2.095	0	↓	1.279	1.258	0.001	↑
Arachidonyl carnitine	549.37614	Pos	1.877	1.875	0	↓	3.891	3.921	0	↑
Cholic acid glucuronide	565.30007	Neg	1.94	1.922	0.006	↓	2.08	2.055	0	↑
CL (8:0/10:0/18:0/18:2(9Z,11Z))	605.39623	Pos	1.584	1.586	0.005	↓	1.859	1.935	0.014	↑
Enterodiol	301.1446	Neg	1.084	1.09	0.007	↓	1.615	1.598	0	↑
FA (18:1(OH3))	329.23343	Neg	1.221	1.219	0	↓	1.427	1.393	0	↑
Gibberellin A88	329.13976	Neg	1.808	1.811	0	↓	1.428	1.442	0	↑
Imidazoleacetic acid ribotide	337.04225	Neg	1.063	1.075	0.006	↓	2.46	2.497	0	↑
Methylnorlichexanthone	271.0614	Neg	1.296	1.318	0.017	↓	1.928	2.016	0.006	↑
Oxindole	134.06007	Pos	1.368	1.365	0	↓	1.024	1.1	0.04	↑
Polyporusterone B	499.30306	Pos	1.193	1.203	0	↓	1.718	1.734	0	↑
Pregnan-20-one, 17-(acetyloxy)-3-hydroxy-6-methyl-, (3b,5b,6a)-	391.28405	Pos	1.455	1.449	0.007	↓	1.768	1.866	0.007	↑
P-Tolyl Sulfate	187.00692	Neg	1.68	1.645	0.009	↓	1.489	1.412	0.029	↑
Valyl-phenylalanine	299.11919	Neg	1.981	1.986	0	↓	1.462	1.498	0	↑

### Bacterial analyses of intestinal contents samples

**Figure 5:**
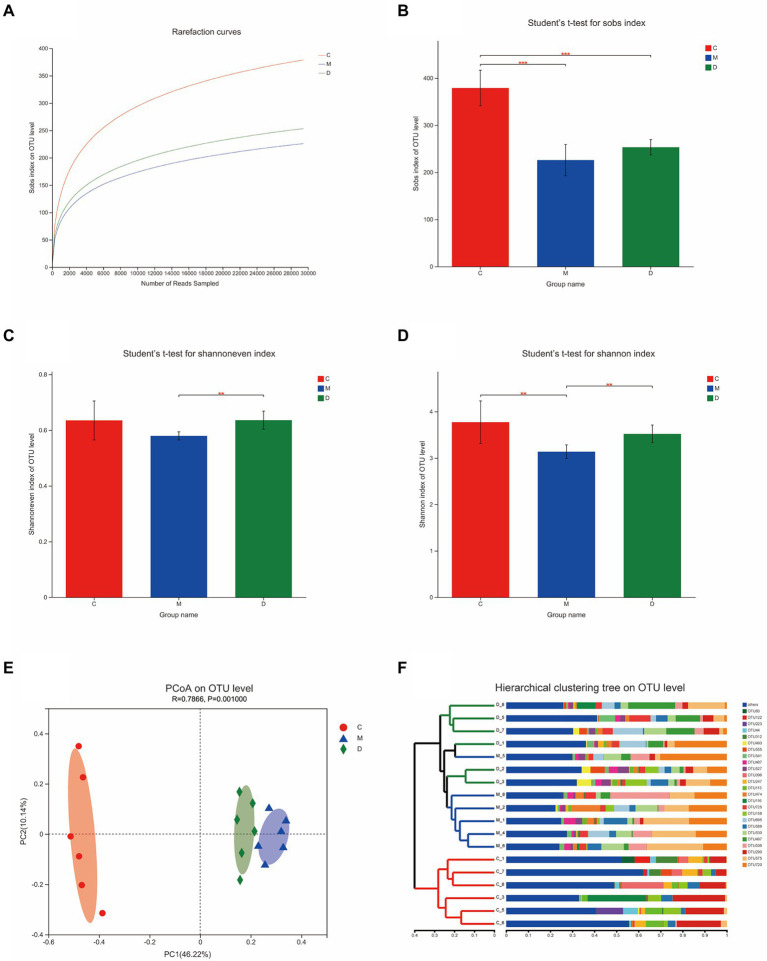
Effects of DBD on the structure and abundance of gut microbiota in T2DM rats. **(A)** Rarefaction curves of 3 group samples. **(B–D)** Alpha diversity is presented by the box plots of Sobs, Shannon, and Shannoneven. **(E)** Principal coordinate analysis (PCoA) plot of the gut microbiota based on Bray–Curtis metrics. **(F)** Hierarchical clustering based on Bray–Curtis distance matrix (C, control; M, model, D-DBD).

**Figure 6:**
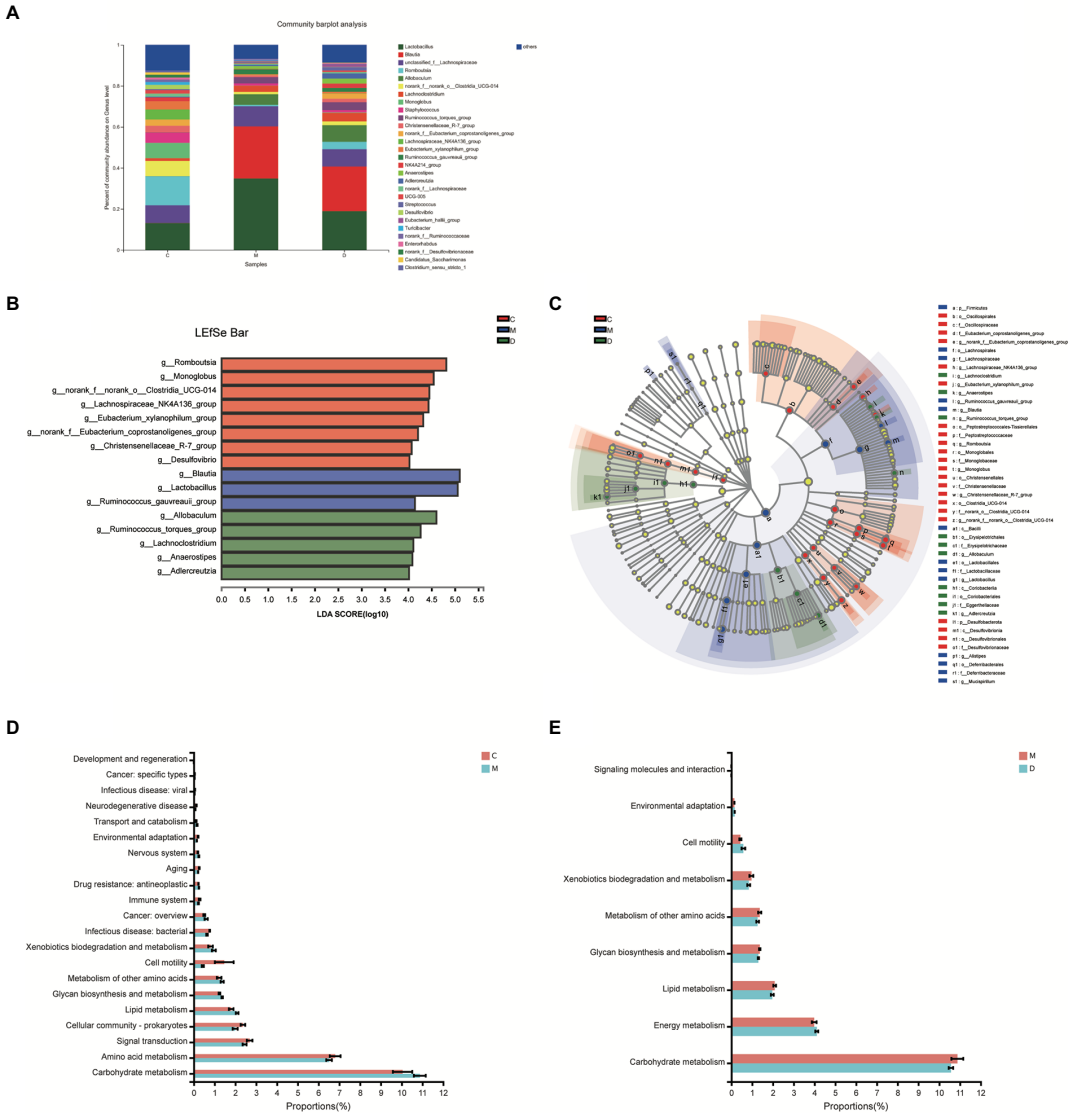
Microbiome analysis of the intestinal contents samples. **(A)** Relative abundance of the gut microbiota at the genus levels. **(B,C)** Histogram and evolutionary branch diagram of LDA distribution between feces in 3 groups. **(D,E)** The proportion of gut microbes in different KEGG pathways with significant differences between the two groups.

In this study, the microbiota composition of the intestinal contents sample was analyzed using 16S rRNA high-throughput sequencing to investigate the potential mechanism of DBD related to gut microbiota. After leveling according to the minimum number of sample sequences, an average of 29,477 sequences per sample was obtained from 24 rat intestinal contents, with 12 phyla, 185 genera, and 725 OTUs. As shown in Figure 5A, the rarefaction curves were gradually smoothed and the Good’s coverage for the observed OTUs was 99.80 ± 0.05%, which indicated that the sample size of this sequencing was sufficient. Besides, the flattening Pan/Core species analysis curve (Supplementary Figure 3) also indicated the sample size sufficient for this sequencing. The Alpha diversity of gut microbiota met a change (Figures 5B,C) and the sob index (*p* < 0.005) and Shannon index (*p* < 0.01) of the model group were lower than that of control which indicated the T2DM significantly decreased community richness and diversity of gut microbiota. After treating with DBD, the Shannon index (*p* < 0.01), and Shannoneven index (*p* < 0.01) of the model group was reversed (Figures 5C,D) that showed a positive regulatory effect on community evenness and diversity. Moreover, the PCoA diagram based on Bray–Curtis distances demonstrated that the microbial community structure was obviously separated between the control group and model group (Figure 5E). The ANOSIM analysis with 999 Monte Carlo random permutations indicated the between-group difference is significantly greater than the within-group difference (R = 0.7866, *p* = 0.001). Besides, the result of the hierarchical clustering tree on OTU level also showed a significant separation in three groups (Figure 5F). On the genus level, obvious difference between microbe relative abundance among these 3 groups could be seen in Figure 6A. After the students *t*-test, the relative abundance of gut microbes in 54 genera was altered between the control group and model group, and 14 genera in the model group and DBD group were changed. The average relative abundance of 8 genera of microbiota in the gut microbes were reversed by DBD, compared to the model group (Supplementary Figures 4A–H). Furthermore, the top 15 species levels of gut microbiota with significant difference between two groups is shown in (Supplementary Figures 4I,J). To further explore differences in the intestinal contents in the gut microbiota among three group, LEfSe multilevel species difference discriminant analysis was used to recognize the specific altered bacterial phenotypes at each phylogenetic level (from phylum to genus, LDA > 4, Figures 6B,C). In total, 11 orders, 12 families, and 17 genera were found to have a significant influence on the differences between groups, most of which belonged to *Firmicutes* and *Desulfobacterota*. The dominant genus in the control group mainly were *Romboutsia*, *Monoglobus*, *norank_f__norank_o__Clostridia_UCG-014*, *Lachnospiraceae_NK4A136_group*, *Eubacterium_xylanophilum_group*, *norank_f__Eubacterium_coprostanoligenes_group*, *Christensenellaceae_R-7_group,* and *Desulfovibrio*. In addition, the *Blautia*, *Lactobacillus,* and *Ruminococcus_gauvreauii_group* had the strongest association with the model group, while *Allobaculum*, *Ruminococcus_torques_group*, *Lachnoclostridium*, *Anaerostipes,* and *Adlercreutzia* were the most representative bacteria in the DBD group. Then, to study the gut microbiome functions, the PICRUSt2 analysis was adopted to analyze the relative abundance of KEGG function in the bacterial community. We found that carbohydrate metabolism, lipid metabolism, glycan biosynthesis, and metabolism of other amino acids, cell motility, xenobiotics biodegradation, and environmental adaptation are the main functions of gut microbiota in cecum, which are reversed following DBD treatment (Figures 6D,E).

### Correlations between biochemical indexes, microbiome, and metabolites in the intestinal contents samples

**Figure 7:**
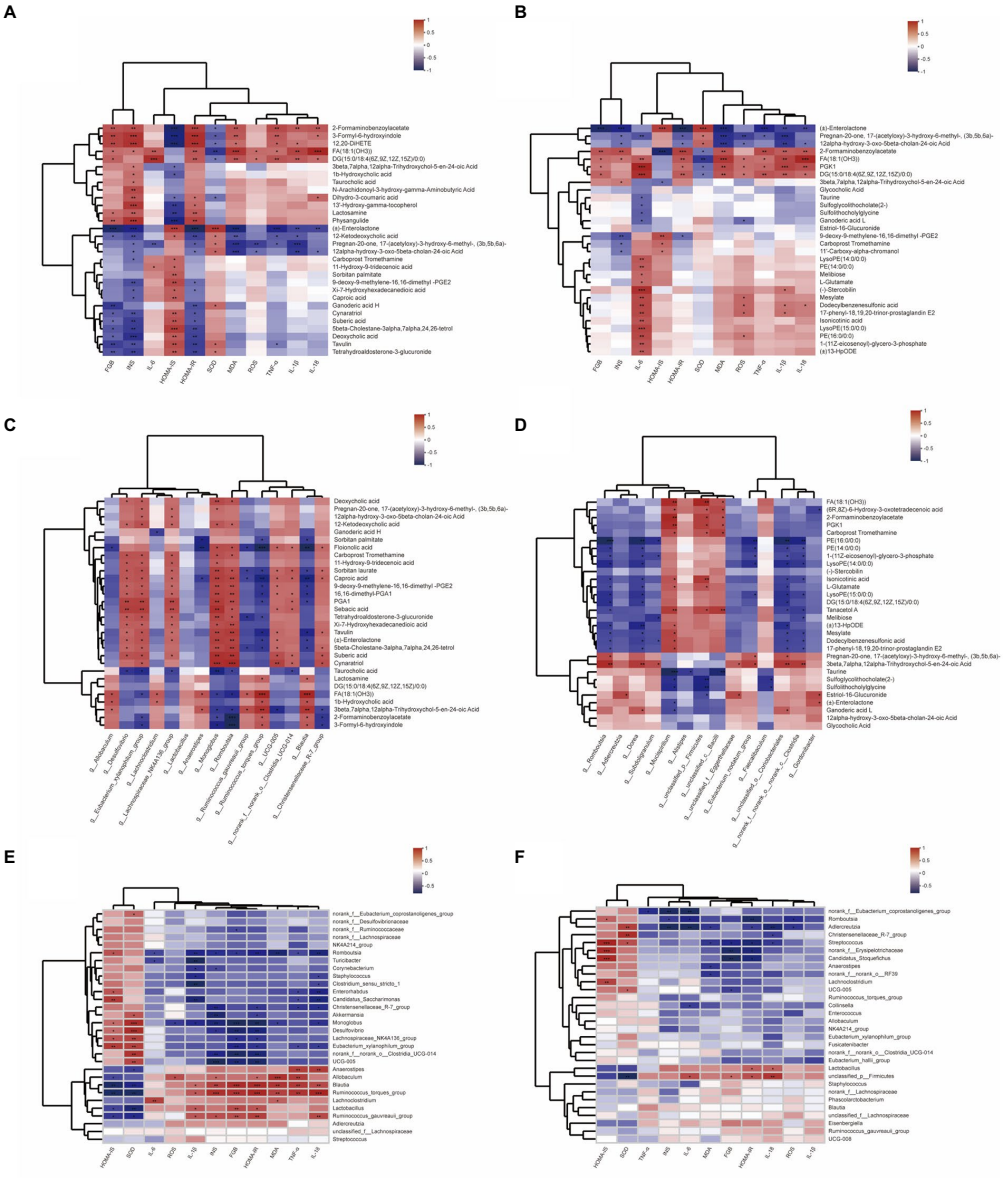
Heatmap of relationships between biochemical indexes, metabolites, and gut microbiota. **(A)** Relationship between biological indicators and the top 30 differential metabolites in control and model group. **(B)** Relationship between biological indicators and the top 30 differential metabolites in model and DBD group. **(C)** Relationship between top 15 differential gut microbiota and the top 30 differential metabolites in control and model group. **(D)** Relationship between top 15 differential gut microbiota and the top 30 differential metabolites in model and DBD group. **(E)** Relationship between biological indicators and the top 15 differential gut microbiota in control and model group. **(F)** Relationship between biological indicators and the top 15 differential gut microbiota in model and DBD group.

To comprehensively analyze the relationships between biochemical indexes, metabolites and gut microbiota, a correlation matrix (heatmap) was established calculating Pearson correlation coefficient, in which the top 15 bacterial species and the top 30 related metabolites in the total metabolites and microbiota with significant differences are analyzed. As shown in Figure 7, the levels of FBG, INS, and HOMA-IR, the pointer of T2DM insulin resistance, have a negative correlation with deoxycholic acid, tetrahydroaldosterone-3-glucuronide, enterolactone, 12-Ketodeoxycholic acid, and other metabolites. Further, enterolactone levels showed a positive relation with *g_Romboutsia* and *g_Gordonibacter*, whose abundance in DBD group was reversed compared to the model group. Besides, 2-Formaminobenzoylacetate, 3-Formyl-6-hydroxyindole, FA (18:1(OH3)), etc. metabolites showed high correlation with biological markers associated with insulin resistance, which played a positive role with *g_unclassified_p__Firmicutes, g_Blautia,* and *g_Mucispirillum*. Inflammation is highly correlated with oxidative stress, and their biochemical indexes were highly positively correlated with the metabolites enriched in the model group, such as PKG1, DG (15:0/18:4(6Z,9Z,12Z,15Z)/0:0), stercobilin, 17-phenyl-18,19,20-trinor-prostaglandin E2, etc., and many of them were also related to insulin resistance, which had a positive relation with *g_Ruminococcus_gauvreauii_group*, *g_Ru*minococcus_torques_group, *g_Blautia, g_Allobaculum*. Moreover, after therapeutic intervention of DBD, the abundance of *g_Romboutsia,* g_Dorea, *g_Blautia, g_Candidatus_Stoquefichus, g_Adlercreutiza,* and other bacterial groups were negatively correlated with inflammatory oxidative factors.

## Discussion

Nowadays, the main treatment options for T2DM, such as sulfonylureas and glinides hypoglycemic agents often have some side effects that cause significant gastrointestinal reactions, hypoglycemia, and weight gain (Flory and Lipska, 2019; Ceriello, 2020). In addition, complications associated with diabetes, for example, diabetic nephropathy, diabetic retinopathy, and diabetic peripheral neuropathy threaten the quality of life of those affected. In the early stage of diabetes, excessive generation of ROS and advanced glycation end products (AGEs) lead to the oxidant response and inflammatory response which play key roles in the pathogenesis of diabetic nephropathy (Singh et al., 2011). TCM compounds composed of a variety of plants have shown excellent potential in the treatment of endocrine diseases, cancer, and many others. Thus, in this study, we focused on the effect of DBD on treatment for T2DM to investigate the potential mechanisms associated with gut microbes and their metabolites of intestinal contents.

The Wistar rat is a kind of closed herd animal and has been widely used in Biomedical experiments. GK rats are non-obese spontaneous type 2 diabetic rats formed by repeated selection inbreeding of Wistar rats (Kitahara et al., 1978), whose pathogenesis, including several susceptibility loci containing genes responsible for some diabetic traits, gestational metabolic impairment inducing an epigenetic programming of the offspring pancreas and the major insulin target tissues, and environmentally induced loss of β-cell differentiation due to chronic exposure to hyperglycemia/hyperlipidemia, inflammation, and oxidative stress have been shown to be highly similar to that of clinical T2DM patients (Portha et al., 2012). Therefore, the GK rat is one of the best characterized animal models of spontaneous T2DM, has proved be a valuable tool offering sufficient commonalities to study T2DM and its complications (Portha et al., 2009). In this experiment, compared with the control group, rats in the model group had obvious symptoms of early insulin resistance in T2DM, such as abnormally elevated serum insulin levels and decreased insulin sensitivity. In addition, with the passage of time, the RGB of the rats in the model group showed a trend towards a continuous increase. After 6 weeks of DBD intervention, the basic biochemical indicators of rats were significantly improved, which was consistent with our previous reports (Wang et al., 2021). In addition, systemic chronic inflammation and oxidative stress are important features in the early stage of type 2 diabetes. One study found that reducing the level of systemic inflammation and oxidative stress can reduce the incidence of diabetic complications (Han et al., 2018). In this experiment, we found that DBD can significantly reduce inflammation and oxidative stress in rat serum, such as TNF-α, IL-18, IL-1β, and MDA. Moreover, in our previous work, we also found that DBD can improve complications such as diabetic nephropathy, diabetic atherosclerosis, and other diseases related to inflammation. Ferulic acid, a natural polyphenol derived from Angelica sinensis, has been found to have significant antioxidant activity. It can improve diabetes insulin resistance through PI3K/AKT, PEPCK, and other signaling pathways (Narasimhan et al., 2015; Cheng et al., 2019). At the same time, a saponin component from Astragalus, Astragaloside IV, has been found to treat diabetes and many of its complications (You et al., 2017; Zhou et al., 2020).

Metabolomics based on LC–MS technology can reveal the physiological and pathological changes of organisms by analyzing the metabolic composition and changes of endogenous and exogenous substances in biological samples, which are of great significance in clarifying the pathogenesis and the treatment of metabolic diseases involving digestion and absorption (De Vos et al., 2022; Pereira et al., 2022). The result of this study shows that DBD can significantly reverse the abundance of 9-OxoODE, enterodiol, enterolactone, arachidonyl carnitine, 2,3-Dinor-6-keto-prostaglandin F1 alpha, 4-OH-Retinal, Nomega-Acetylhistamine, oxindole, pyroglutamylvaline, and other metabolites, which are also changed in model group rats compared to the control group. 9-OxoODE is a kind of product of adipocyte lipolysis oxidized by glutathione peroxidases, which is considered to be of high diagnostic value and helpful in predicting T2DM and its complications (Rhee et al., 2021), and DBD can change the high level of 9-OxoODE. Arachidonyl carnitine, 4-OH-Retinal are involved in fatty acid metabolism and retinol metabolism, the content in the model group rats was significantly reduced, and this phenomenon was also observed in clinical patients with obesity and metabolic-related diseases, which may relate to the excess fatty acid environment and impaired mitochondrial metabolism in overweight or obese patients (Song et al., 2020). Prostacyclin is the main metabolite of arachidonic acid (AA) produced by vascular endothelial cells that has a significant vasodilation effect. Under physiological conditions, prostacyclin cooperates with NO and has a vascular regulation effect, which has poor stability *in vivo* and will spontaneously hydrolyze into 6-keto prostaglandin F1 alpha and its secondary metabolite 2,3-Dinor-6-keto-prostaglandin F1 alpha (Enzler et al., 2012). Diabetes is an important risk factor for cardiovascular disease. In the early stage of microvascular complications of diabetes, vascular endothelial dysfunction is mainly manifested as reduced NO release, and the improvement of vasodilation mediated by prostacyclin helps to compensate to mediate vascular stability, which in turn showed increased levels of 2,3-Dinor-6-keto-prostaglandin F1 alpha (Shi and Vanhoutte, 2017). DBD can reduce the level of 2,3-Dinor-6-keto-prostaglandin F1 alpha in rat intestinal content metabolites, which proves that DBD may help alleviate diabetic microvascular complications and improve microvascular inflammation. N1,N12-Diacetylspermine is an intermediate product of arginine metabolism, which is associated with various diseases such as diabetes, and can be used as a potential biomarker for various malignant tumors (Umemori et al., 2010). The level of N1,N12-Diacetylspermine in the intestinal contents of rats in the model group increased significantly and decreased after intervention with DBD. Phytoestrogens, such as lignans and isoflavone, have been found to have significant anti-inflammatory, anti-oxidative, and tumor-preventive effects (Torrens-Mas and Roca, 2020; Chakraborty et al., 2021). In this experiment, lignans metabolites enterodiol, enterolactone, 2’-Hydroxyenterolactone, among others were significantly decreased in the T2DM model group rats, and the levels of serum inflammatory factors increased, which is consistent with what has been reported in the literature (Sun et al., 2014). *Astragalus* is an important component of DBD, belonging to the *Fabaceae Leguminosae* (*Fabaceae*), rich in lignans, soybean isoflavones, and other phytoestrogens, so the level of lignan metabolites in the DBD group increased, and the serum levels of oxidative inflammatory factors in rats were improved. Therefore, based on the above metabolomic results, we can speculate that the components of DBD can regulate the metabolism of endogenous and exogenous fatty acids, arachidonic acid, amino acids, and phytoestrogens, thereby interfering with the development of T2DM.

In recent years, more and more studies have found that gut microbes are closely related to the occurrence and development of diseases. So, we chose the intestinal contents of the cecum for this study. Changes in the alpha diversity of gut microbes have been shown to be related to diseases, such as extreme obesity and high blood pressure (Wilmanski et al., 2019; Verhaar et al., 2020). In this study, the levels of community richness and the community diversity index of model group rats’ gut microbiota (Sob and Shannon), were decreased significantly. Anfter the intervention of DBD, the levels of gut microbiota’ community evenness and community diversity (Shannoneven and Shannon) were markedly improved. At the phylum level, the abundance of Firmicutes in the intestines of rats in the model group was increased, which is the same as the distribution trend of intestinal bacterial species in patients with T2DM reported in the literature (Massier et al., 2020). From the family level, the higher abundance of *Peptostreptococcaceae* and *Oscillospiraceae* in DBD group rats, which met a significant decrease in the model group, was associated with less T2DM and lower level of serum cholesterol (Maya-Lucas et al., 2019; Chen et al., 2021). *Romboutsia*, a microbiota flora whose abundance was reversed by DBD, has been found to regulate the metabolism of type 1 diabetes rats’ serum and hippocampus to alleviate cognitive dysfunction (Gao et al., 2019). In this experiment, was also found to be closely negatively related to metabolites such as taurocholic acid, which showed a high relation with increased T2DM risk (Lu et al., 2021). Besides, compared with the model group, *Adlercreutzia* increased in the gut microbiota of rats after administration of DBD, and Lucía Vázquez et al. found that *Adlercreutziashu* can hydrolyze daidzein to produce isoflavones (Vázquez et al., 2020a, b). Various glycoside chemical constituents in DBD have been reported in the literature, such as daidzein, calycosin-7-glucoside, and formononetin-7-O-beta-D-glucoside-6-O-malonate (Wen et al., 2007; Liu et al., 2018). That may explain why metabolic pathway analysis found that the products of the isoflavonoid biosynthesis pathway were significantly enriched in the intestinal contents of rats in the DBD group. Ting Hu et al. found that, compared with glycosides, flavonoids such as calycosin and other flavonoids can exert greater antioxidant and anti-inflammatory effects (Yu et al., 2005; Hu et al., 2017). So, as reported in the literature, the involvement of bacterial metabolism may play a positive role in the beneficial effects of phytoestrogens on human health (Peirotén et al., 2020).

## Conclusion

In general, DBD extract can improve insulin resistance, systemic inflammation and oxidative stress, and regulate metabolic profiles in T2DM model rats. Based on the metabolome and 16S rRNA technology, the mechanism of its anti-inflammatory and antioxidant effect may be associated with the regulation of metabolites of endogenous and exogenous fatty acids, arachidonic acid, amino acids and phytoestrogens, as well as the reversion of intestinal flora disorders caused by T2DM. To confirm the regulation of DBD on the metabolism and gut microbiome, the key pathways still need to be verified by further molecular biology experiments. The results may provide new ideas and insights for the treatment of T2DM in the clinic and be helpful for development of new therapeutic drugs.

## Data availability statement

The datasets presented in this study can be found in online repositories. The names of the repository/repositories and accession number(s) can be found at: https://www.ncbi.nlm.nih.gov/, PRJNA830524.

## Ethics statement

The animal study was reviewed and approved by Committee on the Ethics of Animal Experiments of Nanjing University of Chinese Medicine and Experimental Animal Welfare of Nanjing University of Chinese Medicine, China (no. 012071002558).

## Author contributions

W-kW and LF: methodology, investigation, formal analysis, and writing—original draft preparation. FG: methodology and writing—reviewing and editing. ZL and JZ: data curation and investigation. KY and JX: validation and data curation. MX: supervision, project administration, and funding acquisition. All authors contributed to the article and approved the submitted version.

## Funding

This study was supported by the National Natural Science Foundation of China (no. 81904085), Nanjing University of Chinese Medicine Foundation Youth Project (NZY81904085), Jiangsu University Students Innovation and Entrepreneurship Training Program project (103152021003).

## Conflict of interest

The authors declare that the research was conducted in the absence of any commercial or financial relationships that could be construed as a potential conflict of interest.

## Publisher’s note

All claims expressed in this article are solely those of the authors and do not necessarily represent those of their affiliated organizations, or those of the publisher, the editors and the reviewers. Any product that may be evaluated in this article, or claim that may be made by its manufacturer, is not guaranteed or endorsed by the publisher.

## Footnotes

^1^ http://www.hmdb.ca

^2^ https://metlin.scripps.edu/

^3^ https://www.majorbio.com

^4^ http://drive5.com/uparse/
