# Supplementary figures and images for: Effects of Danggui Buxue decoction on host gut microbiota and metabolism in GK rats with type 2 diabetes
Source: Front Microbiol. 2022 Oct 24;13:1029409. doi: 10.3389/fmicb.2022.1029409 (PMC9638067; doi:10.3389/fmicb.2022.1029409)

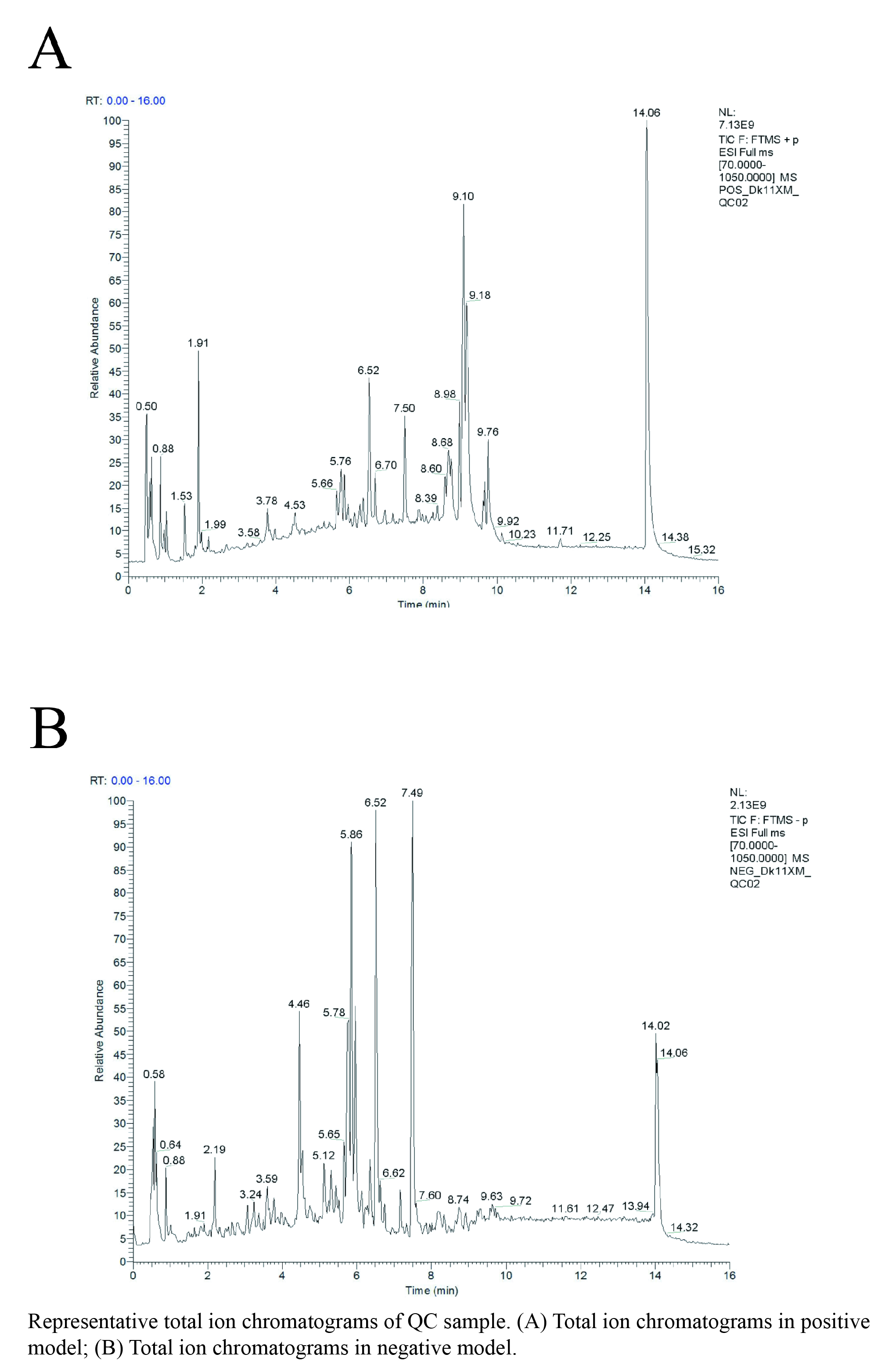

Supplement: Supplementary file 1 [file Image_1.jpg]

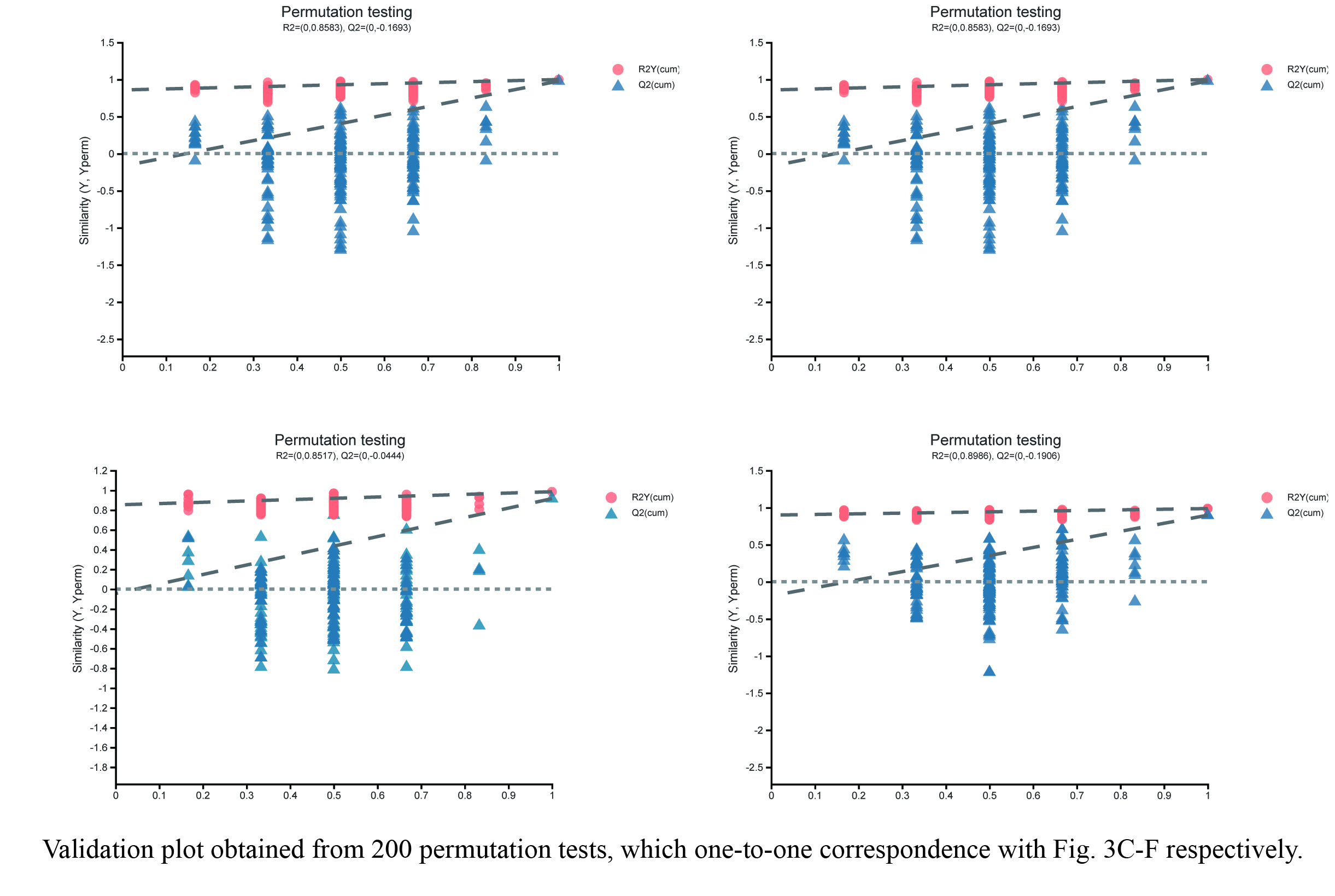

Supplement: Supplementary file 2 [file Image_2.jpg]

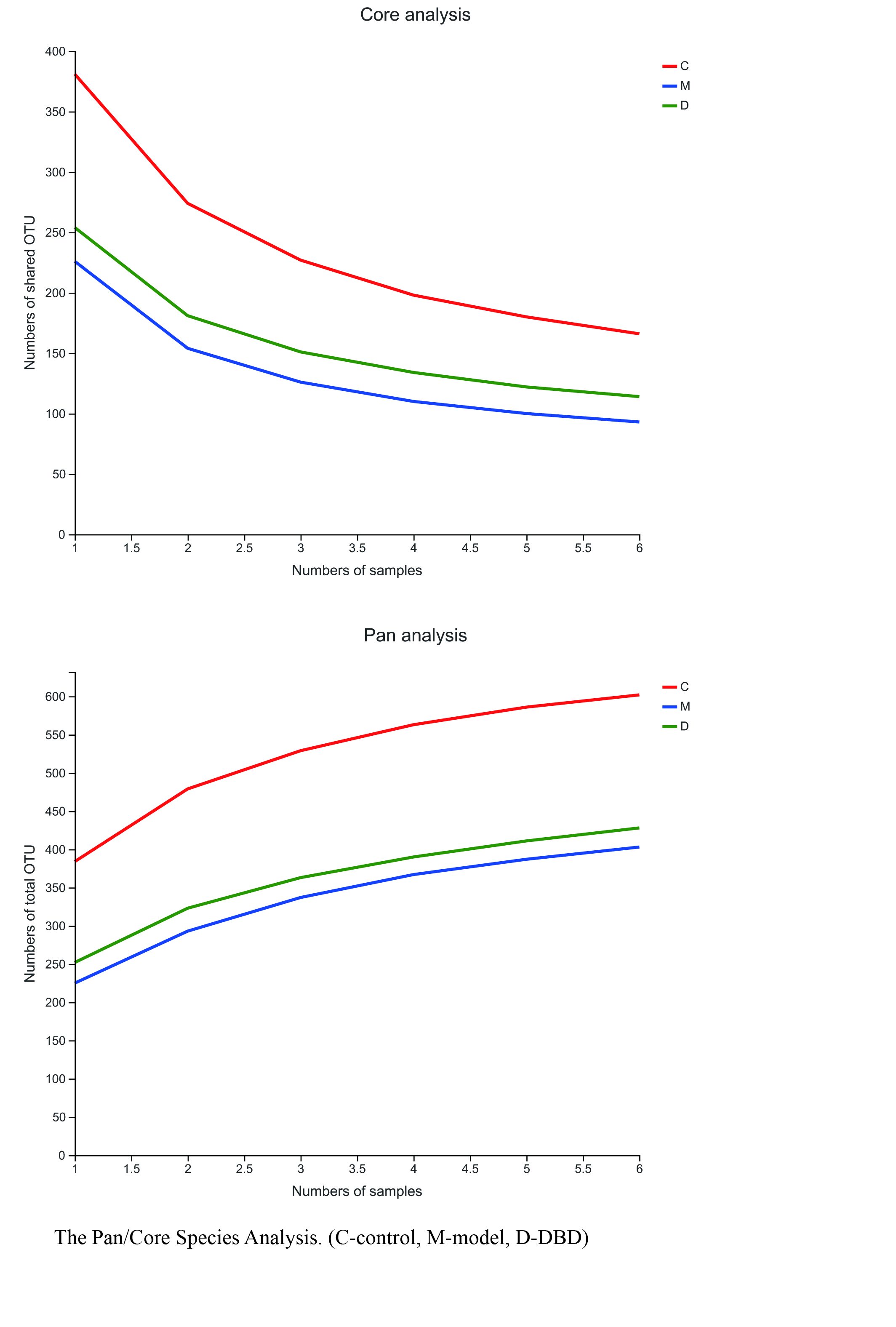

Supplement: Supplementary file 3 [file Image_3.jpg]

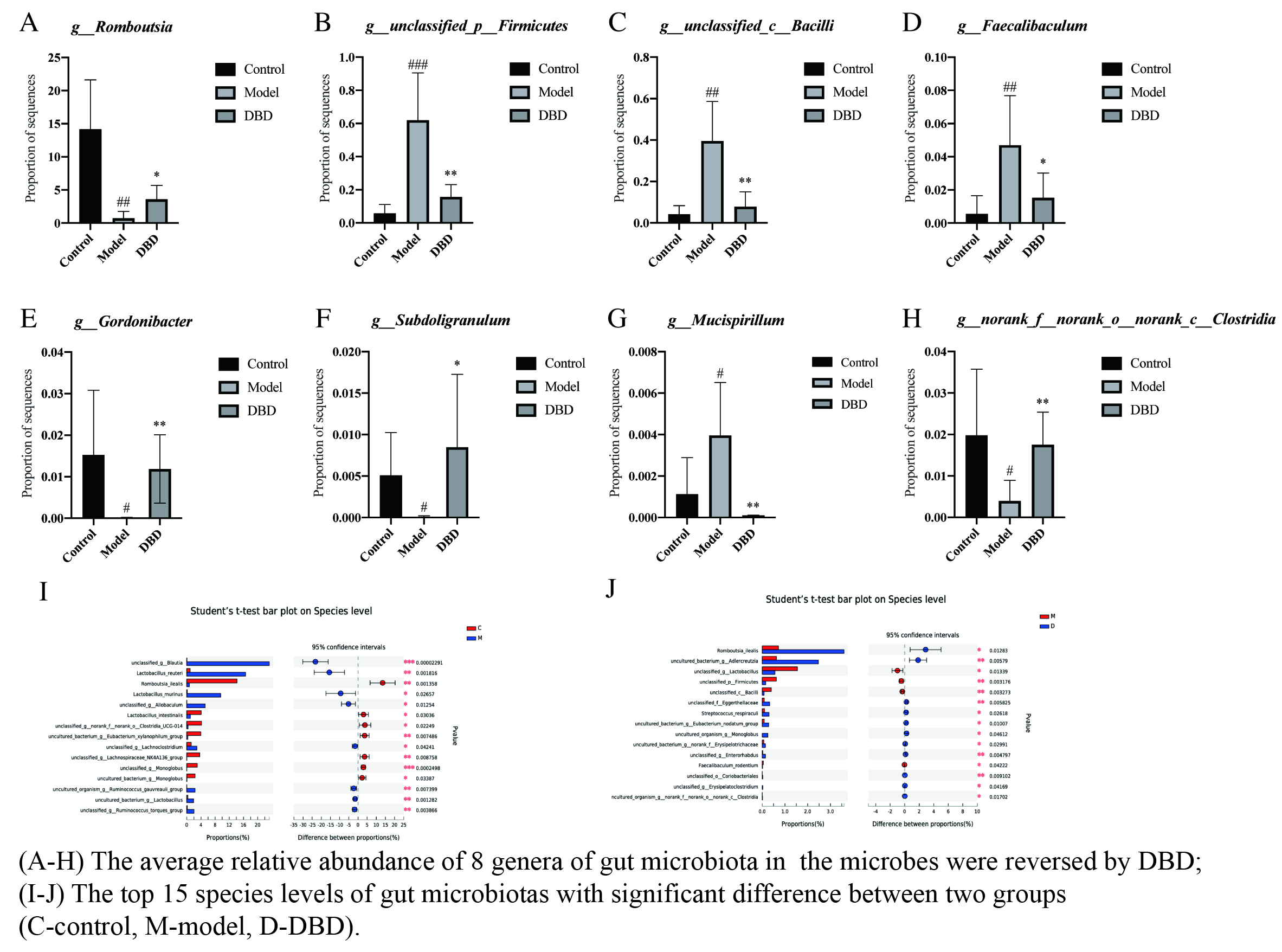

Supplement: Supplementary file 4 [file Image_4.jpg]
